# All-optical recording and stimulation of retinal neurons *in vivo* in retinal degeneration mice

**DOI:** 10.1371/journal.pone.0194947

**Published:** 2018-03-29

**Authors:** Soon Keen Cheong, Jennifer M. Strazzeri, David R. Williams, William H. Merigan

**Affiliations:** 1 Center for Visual Science, University of Rochester, Rochester, New York, United States of America; 2 Flaum Eye Institute, University of Rochester, Rochester, New York, United States of America; 3 Institute of Optics, University of Rochester, Rochester, New York, United States of America; University of California Berkeley, UNITED STATES

## Abstract

Here we demonstrate the application of a method that could accelerate the development of novel therapies by allowing direct and repeatable visualization of cellular function in the living eye, to study loss of vision in animal models of retinal disease, as well as evaluate the time course of retinal function following therapeutic intervention. We use high-resolution adaptive optics scanning light ophthalmoscopy to image fluorescence from the calcium sensor GCaMP6s. In mice with photoreceptor degeneration (rd10), we measured restored visual responses in ganglion cell layer neurons expressing the red-shifted channelrhodopsin ChrimsonR over a six-week period following significant loss of visual responses. Combining a fluorescent calcium sensor, a channelrhodopsin, and adaptive optics enables all-optical stimulation and recording of retinal neurons in the living eye. Because the retina is an accessible portal to the central nervous system, our method also provides a novel non-invasive method of dissecting neuronal processing in the brain.

## Introduction

The development of vision restoration therapies such as optoelectronic, optogenetic, gene therapy, and stem cell therapies [1–5] is slow because of limitations in existing methods to evaluate the efficacy of the restoration. Methods such as patch clamp and multi-electrode array recording provide single-cell resolution but only at a single time point per animal because they are *ex vivo* techniques. *In vivo* methods such as electroretinography and visual evoked potential recording can be used for longitudinal studies but do not provide information about changes in individual retinal neurons. The gold standard for evaluating efficacy of vision restoration is inevitably psychophysical but it is time consuming and usually ill-suited to determine why a particular method fails. An imaging method that records the responses of many individual neurons in the intact eye could accelerate the development of novel vision restoration therapies by allowing direct and repeatable visualization of cellular function over time throughout the therapeutic process.

We previously demonstrated proof of concept of functional adaptive optics cellular imaging in the living eye (FACILE) in both the mouse [6] and monkey [7]. It combines high-resolution adaptive optics scanning light ophthalmoscopy (AOSLO) [8, 9] with the genetically encoded calcium sensor GCaMP6s [10] for single cell recording in the living eye. Here we detail significant improvements to FACILE, including achieving consistent widespread expression of GCaMP6s in mice, and implementation of Fourier analyses for efficiently extracting responses. We demonstrate the ability to measure the responses of hundreds of individual retinal neurons with high sensitivity. Indeed, we demonstrate that our method is sensitive enough to detect light driven neuronal responses in *rd10* mice at ages where visual responses are undetectable using electroretinogram [11–13]. We demonstrate the use of FACILE to monitor the success of an optogenetics strategy for vision restoration. Among the variety of approaches being developed to restore vision we chose to study optogenetics because of its potential to restore vision (for example [14–17]) in a wide variety of retinal diseases and the simplicity of administration, which requires a single intravitreal injection. We used the red-shifted channelrhodopsin ChrimsonR [18] in this study for two reasons. The first is because the longer wavelength light needed to activate ChrimsonR is less phototoxic than short wavelength light [19]. The second is that ChrimsonR mediated responses are easy to distinguish from those of the blue-green sensitive intrinsically-photosensitive ganglion cells that survive in photoreceptor degenerative diseases, as well as any response that may be mediated by residual photoreceptors [20]; the loss of photoreceptors in photoreceptor degenerative diseases is often incomplete [21–23]. We employed the *rd10* mouse model of retinal degeneration because it closely mimics the cause and pathogenesis of human photoreceptor degenerative disease [11, 24], for example retinitis pigmentosa. In *rd10* mice treated with ChrimsonR, we measured and tracked restored visual responses in retinal neurons over a six-week period following significant loss of visual responses. This study provides direct evidence that the light responses of individual retinal neurons restored by ChrimsonR remain robust over an extended period of time.

By combining a fluorescent calcium sensor, a channelrhodopsin, and AOSLO we show that it is possible to establish an all-optical [25] ‘read-write’ interface with large numbers of retinal neurons *in vivo*. Because the retina is an accessible portal to the central nervous system, our novel method also provides a non-invasive tool to disentangle complex neural circuitry and processing of the brain.

## Materials and methods

All animal procedures were conducted according to the ARVO Statement for the Use of Animals in Ophthalmic and Vision Research. All protocols were approved by the University of Rochester Committee on Animal Resources.

### Animal preparation

Adult photoreceptor degeneration type 10 (*rd10*, strain B6.CXB1-Pde6b^*rd10*^/J, The Jackson Laboratory, USA) and wild-type (WT, C57BL/6J, The Jackson Laboratory, USA) mice were used in this study. They were house in standard cages, up to five individuals per cage, on a 12 hour light-dark cycle. Animals were not used for any prior experiments. For intravitreal injections and fundus imaging (< 30 min), mice were anesthetized using ketamine (0.1 mg g^-1^, JHP Pharmaceuticals, USA) and xylazine (0.01 mg g^-1^, Akorn Inc., USA). For adaptive optics (AO) imaging (~ 2 h), mice were anesthetized using a cocktail containing fentanyl (0.05 μg g^-1^, West-Ward Pharmaceuticals Corp., USA), dexmedetomedine (Dexdomitor, 0.5 μg g^-1^, Orion Corp., Finland), and midazolam (5 μg g^-1^, West-Ward Pharmaceuticals Corp., USA). At the conclusion of the AO imaging session mice were given a reversal cocktail containing naloxone (1.2 μg g^-1^, Hospira Inc., USA), atipamezole (Antisedan, 2.5 μg g^-1^, Orion Corp., Finland), and flumazenil (0.5 μg g^-1^, Hikma Pharmaceuticals, UK). All drugs were administered intraperitoneally. Adequate anesthesia was evaluated by checking the toe-pinch and corneal reflexes. During AO imaging, additional heating was provided to maintain the internal body temperature at 37°C and animals were ventilated on 100% oxygen. Ophthalmic hypromellose gel (Genteal, 0.3%, Alcon, USA) was applied to prevent the corneas from drying. Antibacterial steroidal ophthalmic ointment (Neomycin and Polymyxin B Sulfates, Bacitracin Zinc with Hydrocortisone Ophthalmic Ointment USP, Bausch and Lomb, USA) was applied at the end of each imaging procedure to prevent the development of corneal opacities due to infection and drying.

### AAV mediated gene transfection of mouse retinal neurons

Adeno-associated viral (AAV) vectors were used to transfect mouse retinal neurons with either GCaMP6s, or ChrimsonR, or both. Constructs used were Syn.ChrimsonR-tdTomato.WPRE.bGH and Syn.GCaMP6s.WPRE.SV40 packaged in either AAV2 or AAV9 capsids. Viruses were purchased as custom preparations from the University of Pennsylvania Vector Core. Viral titers were 1.92x10^13^ GC/mL for AAV2.ChrimsonR, 2.23x10^13^ for AAV9.ChrimsonR, 2.38x10^13^ GC/mL for AAV2.GCaMP6s, and 4.04x10^13^ for AAV9.GCaMP6s. Intravitreal injections were made in animals that were at least 3 weeks of age.

**Fig 1:**
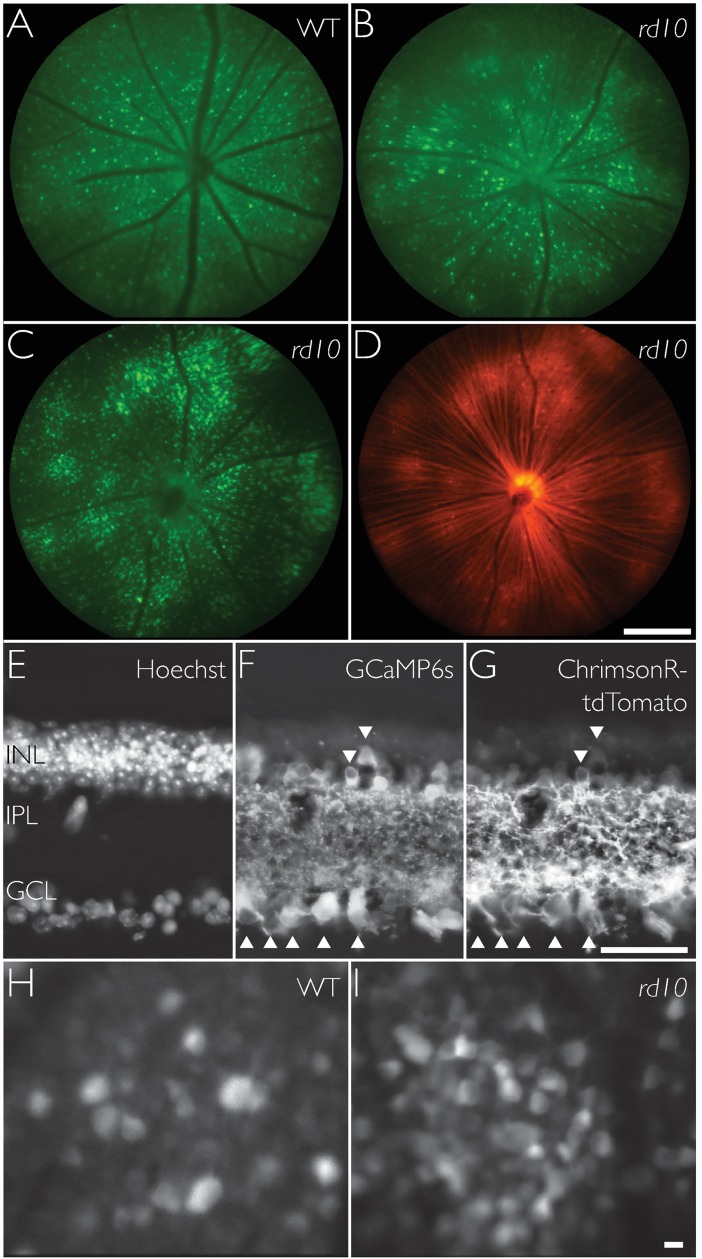
Expression of GCaMP6s and ChrimsonR-tdTomato in mouse retinae. (A-D) Fluorescent fundus images showing (A) GCaMP6s expression in wild-type (WT) mouse retina at age P64, 20 days after injection of AAV2.GCaMP6s, (B) GCaMP6s expression in *rd10* mouse retina not treated with ChrimsonR at age P43, 19 days after injection, and (C & D) GCaMP6s expression and ChrimsonR-tdTomato co-expression in *rd10* mouse retina at age P93, 49 days after injection of AAV2.GCaMP6s and AAV2.ChrimsonR. Scale bar in D indicates 500 μm. (E-G) Vertical section of an *rd10* retina showing Hochst-stainned nuclei (E), expression of GCaMP6s (F) and ChrimsonR-tdTomato (G). Cell bodies co-labeled with GCaMP6s and ChrimsonR are indicated by arrows. Tissue was harvested from a mouse aged P240, 196 days after injection. Scale bar in G indicates 50 μm. (H & I) GCaMP6s fluorescent neurons in a WT mouse at age P50, 26 days after injection, (H) and *rd10* mouse treated with ChrimsonR at age P112, 69 days after injection, (I) imaged *in vivo* using an adaptive optics ophthalmoscope focused at the ganglion cell layer. Scale bar in I indicates 10 μm.

Achieving consistent robust expression of both GCaMP6s and ChrimsonR was a critical step in being able to perform FACILE. We initially used GCaMP6s packaged in an AAV9 capsid, which yielded inconsistent expression. In WT mice, expression was observed in 72.8% of eyes injected with the AAV9 virus (n = 66). Expression was weak and/or patchy. In comparison, with an AAV2 virus, expression of GCaMP6s was observed in 93.7% of eyes injected (n = 16), and was generally robust and widespread (Fig 1A). In *rd10* mice, a similar trend was observed. With AAV9, expression was observed in 53.2% of eyes injected (n = 47), and with AAV2 100% of eyes injected (n = 39) showed expression of GCaMP6s (Fig 1B and 1C). We also used AAV9.Syn.ChrimsonR-tdTomato and found that expression was generally weak and limited to the region surrounding the optic disk. In sum, expression of GCaMP6s and ChrimsonR in inner retinal neurons was best achieved with AAV2 capsid delivered using intravitreal injection.

We initially made serial injections of GCaMP6s and then ChrimsonR with the aim to first evaluate the loss of photoreceptor mediated visual responses in *rd10* mice and then record restored visual activity mediated by ChrimsonR. In 10 *rd10* eyes we first injected GCaMP6s and then ChrimsonR 48 days later. All eyes showed robust expression of GCaMP6s but only half showed subsequent expression of ChrimsonR. In another 10 *rd10* mouse eyes we performed the converse experiment, first injecting ChrimsonR then GCaMP6s 21 days later. We observed robust widespread expression of ChrimsonR in 8 eyes but no GCaMP6s expression in any eye. Finally, we made co-injections of 1 μl of GCaMP6s and 1 μl of ChrimsonR. Expression of both GCaMP6s and ChrimsonR was observed in 9 eyes; 1 eye was not imageable. These observations suggest that mice build an immune response to AAVs that limits the success of serial injections that are weeks apart. In sum, co-expression was best achieved with co-injections.

For the results we report here, four female WT mice, four male and one female *rd10* mice received only GCaMP6s (2 μl per eye). Three female *rd10* mice received both GCaMP6s and ChrimsonR-tdTomato (1 μl per virus per eye). A fundus camera (Micron III, Phoenix Research Laboratories, USA) with custom filters (GCaMP6s: excitation FF01-498 SP [Semrock Inc, USA], emission ET525/50 [Chroma Technology Corporation, USA]; ChrimsonR-tdTomato: excitation FF02-525/40 [Semrock Inc, USA], emission band-pass 590/47) was used to assess and map expression of GCaMP6s and ChrimsonR-tdTomato. The scale of fundus images was measured by matching vascular landmarks, such as blood vessel branch points, in an adult WT mouse retina in fundus images and in micrographs taken of the same retina, unfixed, with a calibrated microscope.

### *In vivo* functional adaptive optics calcium imaging

High resolution *in vivo* retinal imaging was performed using a custom built mouse adaptive optics scanning light ophthalmoscope (see [26] for system details). All *in vivo* imaging was performed within an eccentricity of 20˚ from the center of the optic disk. Fluorescence from GCaMP6s was excited using a 488 nm laser diode (iChrome MLE-L, Toptica Photonics Inc., USA) and band-pass filtered (FF01–520/35, Semrock Inc, USA). Simultaneous reflectance imaging of blood vessels was performed using a 790 nm laser diode (S790-G-I-15, Superlum Diodes Ltd., Ireland). Eye motion was computed using the reflectance images of retinal vasculature [27] and motion correction was applied to both reflectance and fluorescence data. Wavefront correction was done using a 905 nm laser diode (QFLD-905-10S, QPhotonics LLC, USA) and deformable mirror. All imaging lights were scanned over a 5 x 6.7° (160 x 215 μm) field on the retina. Light intensity at pupil was 100 μW for 488 nm, 185 μW for 796 nm, and 7 μW for 904 nm. A 75 μm pinhole for was used for fluorescence imaging and 50 μm pinhole infrared reflectance imaging.

**Fig 2:**
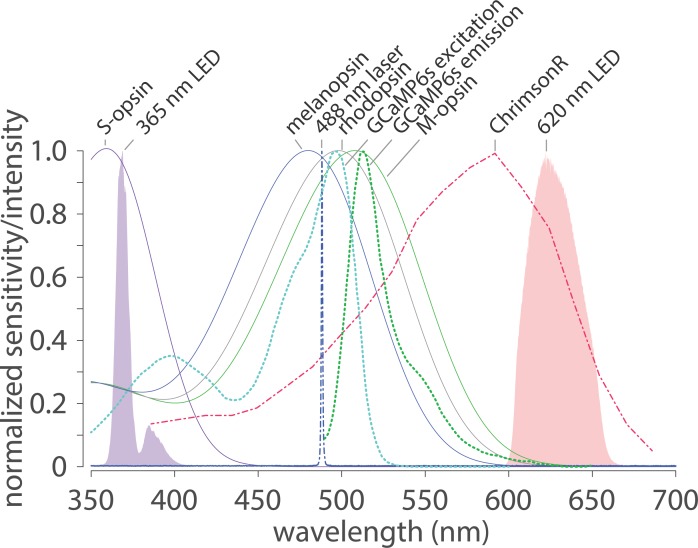
Action spectra of mouse photoreceptors, GCaMP6s, ChrimsonR, and light sources for FACILE. Normalized action spectra of mouse photoreceptor opsins, melanopsin, ChrimsonR, GCaMP6s excitation and emission, and measured spectra of light sources: 488 nm GCaMP6s excitation laser, and 365 nm and 620 nm stimulating LEDs.

Mice were positioned in a custom-built holder with bite bar. Mydriasis and cyclopegia were achieved with one drop of phenylephrine hydrochloride (2.5%, Akorn Inc., USA) and one drop of tropicamide (0.5%, Alcon, USA). Contact lenses (material: PMMA, base curvature: 1.6 mm, power: +10 D, diameter: 3.0 mm, center thickness: 0.3, Advanced Vision Technologies, USA) were placed on the eyes using lubricant eye drops (carboxymethylcellulose sodium 0.5%, Refresh Tears, Allergan, USA) to prevent the corneas from drying. LEDs with peak emission at 365 nm (M365L2-UV, Thorlabs, USA) to drive short wavelength sensitive (S) opsin, or 620 nm (M565L3, Thorlabs, USA; with band-pass filter FF01–571/72–25, Semrock Inc, USA) to drive ChrimsonR were used to provide visual stimuli in Maxwellian view over 8° diameter patch of retina. Light power of the LEDs measured at the pupil were 20 μW for 365 nm, and 100 μW for 620 nm. Retinal irradiance was calculated to be 33.6 mW.cm^-2^ for 365 nm, and 168.1 mW.cm^-2^ for 620 nm. Stimuli were temporally modulated (0.2 Hz), uniform field, square waves. Spectra of light sources used in AO imaging were measured using a spectrometer (USB4000, Ocean Optics, USA). Fig 2 shows the measured spectra of the stimulation and imaging lights, spectral sensitivities of mouse opsins, and action spectra of GCaMP6s.

**Fig 3:**
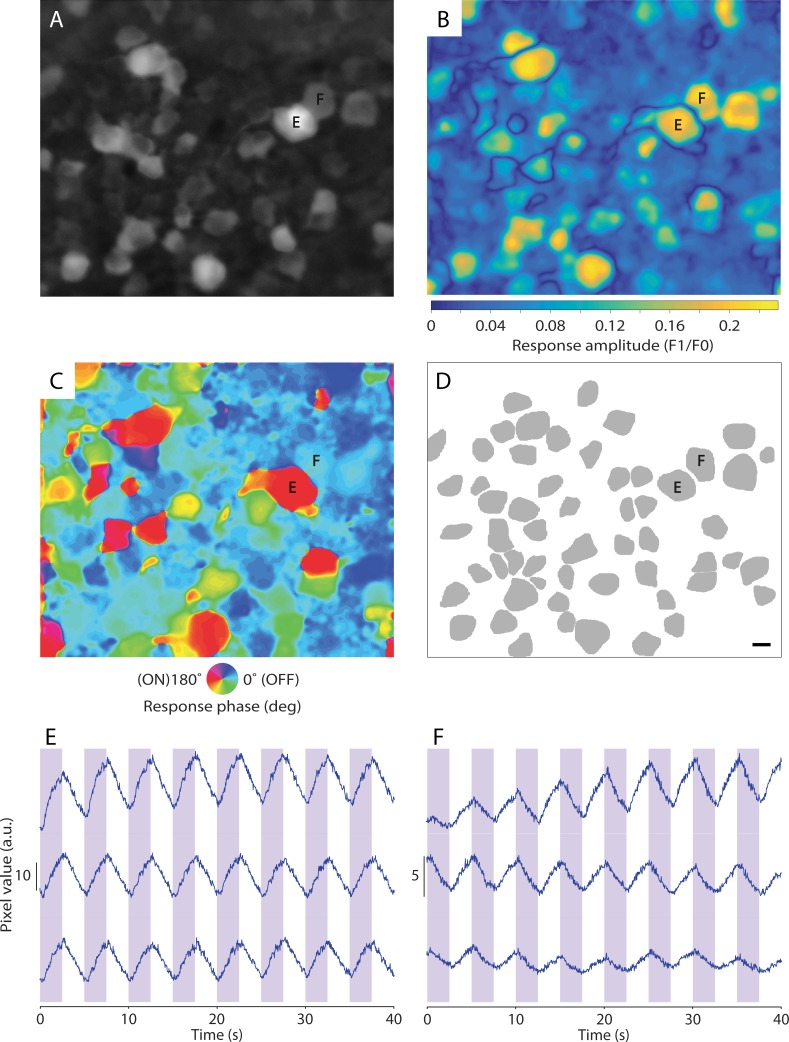
Computing neuronal responses from *in vivo* calcium imaging data. (A) GCaMP6s fluorescent retinal neurons in a WT mouse retina imaged *in vivo* using an adaptive optics ophthalmoscope focused at the ganglion cell layer. (B) Color map showing normalized response amplitude to the UV (365 nm) stimulus. (C) Color map of response phase to the 365 nm stimulus. (D) Cell segmentation mask constructed from fluorescence intensity image (B) and response color maps (C & D). Scale bar indicates 10 μm and applies to A-D. (E & F) Response time courses of cells indicated in A-D. Shaded bars indicate presentation of the 365 nm LED at 20 μW. Three consecutive trials are shown, the first at the top. Cell E shows ON responses to UV light. Cell F shows OFF responses to UV light.

Neuronal responses, measured using GCaMP6s, were quantified by computing the power and phase of the GCaMP6s fluorescent signal at the temporal frequency of the stimulus (fundamental harmonic or F1). A normalized response was computed by dividing the F1 by the mean of the response time course (F0). Activity maps of normalized response amplitude (F1/F0, Fig 3B) and phase (Fig 3C) were constructed by applying the frequency computation to the video sequence on a pixel-by-pixel basis. Cell segmentation was performed manually to generate an ROI mask (Fig 3D) using activity maps and SUM image (Fig 3A), constructed by integrating all frames in the video. The ROI mask was applied to the original video sequence and average response time courses were computed for each ROI (Fig 3E and 3F). Response for each cell was then quantified using the frequency analysis described above. Recording noise was analyzed by computing the mean and SD amplitude of response from 10–12 Hz. All data analysis was done using MATLAB (ver. 2015b, MathWorks, USA).

### Histology

Animals were euthanized using CO2 asphyxiation and cervical dislocation. Eyes were immediately enucleated and immersed in 4% paraformaldehyde in 0.1M phosphate buffer, pH 7.4, for 1–2 hours. The anterior chamber, lens and vitreous were removed. Transverse retinal sections of 12 μm thickness were cut using a cryostat and stained with Hoechst 33342 (0.5 μg ml^-1^, Thermo Fischer Scientific, USA) for 10 minutes then washed in phosphate buffer for 10 minutes. Sections were mounted using antifade mounting medium (Vectashield, Vector Laboratories, USA). Images were taken with a Zeiss M1 epifluorescence microscope with AxioVision software.

## Results

### Robust, widespread expression of GCaMP6s and ChrimsonR in retinal neurons

To measure neuronal responses to light, the genetically encoded calcium sensor GCaMP6s was inserted into wild-type (WT, C57BL/6J) mice and *rd10* (B6.CXB1-Pde6b^*rd10*^/J) mice by intravitreal injection of AAV2.Syn.GCaMP6s. Fig 1A and 1B show fluorescence fundus images of GCaMP6s expression in a WT and *rd10* mouse retina, respectively. To study channelrhodopsin mediated restored vision, another group of *rd10* mice received co-injections of AAV2.Syn.ChrimsonR-tdTomato and AAV2.Syn.GCaMP6s. Fig 1C and 1D show expression of GCaMP6s and tdTomato tagged ChrimsonR respectively in the same *rd10* mouse retina. Note the substantial axonal label in Fig 1D indicating expression of ChrimsonR in ganglion cells. Because ChrimsonR is a membrane-localized ion channel, the axon bundles appear more prominently labelled, as opposed to somata, as there is relatively more membrane to cytoplasm in the axon versus the cell body. Conversely, GCaMP6s expression is cytosolic, and thus more prominently labels cell bodies as there is relatively more cytosol in the cell body than in the axon. Despite our attempt at maintaining consistent injection parameters, we observed varying expression pattern (patchiness) and intensity between eyes, which we attribute to inherent variability between eyes. Fig 1E–1G show histological micrographs of *rd10* retina that received both GCaMP6s and ChrimsonR. Cell nuclei stained with Hoechst (Fig 1E) indicate the ganglion cell (GCL), inner plexiform (IPL), and inner nuclear (INL) layers. The outer nuclear and outer plexiform layers are not present because the photoreceptors have degenerated [11–13, 24]. Cytoplasmic expression of GCaMP6s was observed in the soma of cells in the GCL as well as in some cells in the INL, and processes in the IPL (Fig 1F). Membrane-localized expression of ChrimsonR-tdTomato was observed in cell bodies in the GCL and INL (Fig 1G); arrows indicate co-labeled cells. The IPL was strongly labeled with ChrimsonR-tdTomato because of the high density of cell membranes. Fig 1H and 1I show images of GCaMP6s fluorescent neurons imaged *in vivo* using an AOSLO in a WT and *rd10* mouse retina respectively. The plane of focus was at the ganglion cell layer. Nuclei can be seen as darkened regions within some cell bodies, consistent with the cytoplasmic expression of GCaMP6s. Our AOSLO instrument has sufficient resolution to section the ganglion cell layer from the rest of the retina. The use of a synapsin promoter limits the expression of GCaMP6s and ChrimsonR to neurons but is not subtype specific. Thus, AO images of neurons in the GCL are likely to contain displaced amacrine cells, which are estimated to make up ~59% of neurons in the GCL [28].

### Functional adaptive optics cellular imaging in the living eye

Fig 2 shows the measured spectra of the light sources used in FACILE along with the action spectra of mouse photoreceptors [29–31], GCaMP6s [32], and ChrimsonR [18]. A 488 nm laser was used to excite GCaMP6s fluorescence. For visual stimulation, a 365 nm ultraviolet (UV) LED was used to stimulate mouse short-wavelength sensitive (S) opsin to drive intrinsic visual responses. A 620 nm red LED was used to drive ChrimsonR. A band-pass filter (FF01–520/35, Semrock Inc, USA) (S1 Fig) was used to transmit light from GCaMP6s fluorescence as well as block light from tdTomato fluorescence and the stimulating LEDs. All *in vivo* imaging was performed in central retina within an eccentricity of 20°.

Because FACILE requires exposing the retina to high intensity visible light, damage can occur to the retina due to phototoxicity [33, 34]. Improved efficiency in extracting visual responses reduces the total number of trials needed and therefore the time the retina is exposed to light. To this end, we implemented a Fourier analysis technique routinely used in visual electrophysiology [35]. The classical method to quantify calcium responses from fluorescent calcium indicators is to compute the ratio of fluorescence change commonly referred to as ΔF/F. This metric is not ideal for functional imaging of retinal neurons for two reasons. First, the light used to excite the fluorescent calcium indicator activates the visual response. Second, spontaneous activity may be falsely interpreted as a response to visual stimulation; spontaneous activity is known to increase during retinal degeneration [36–38].

**Fig 4:**
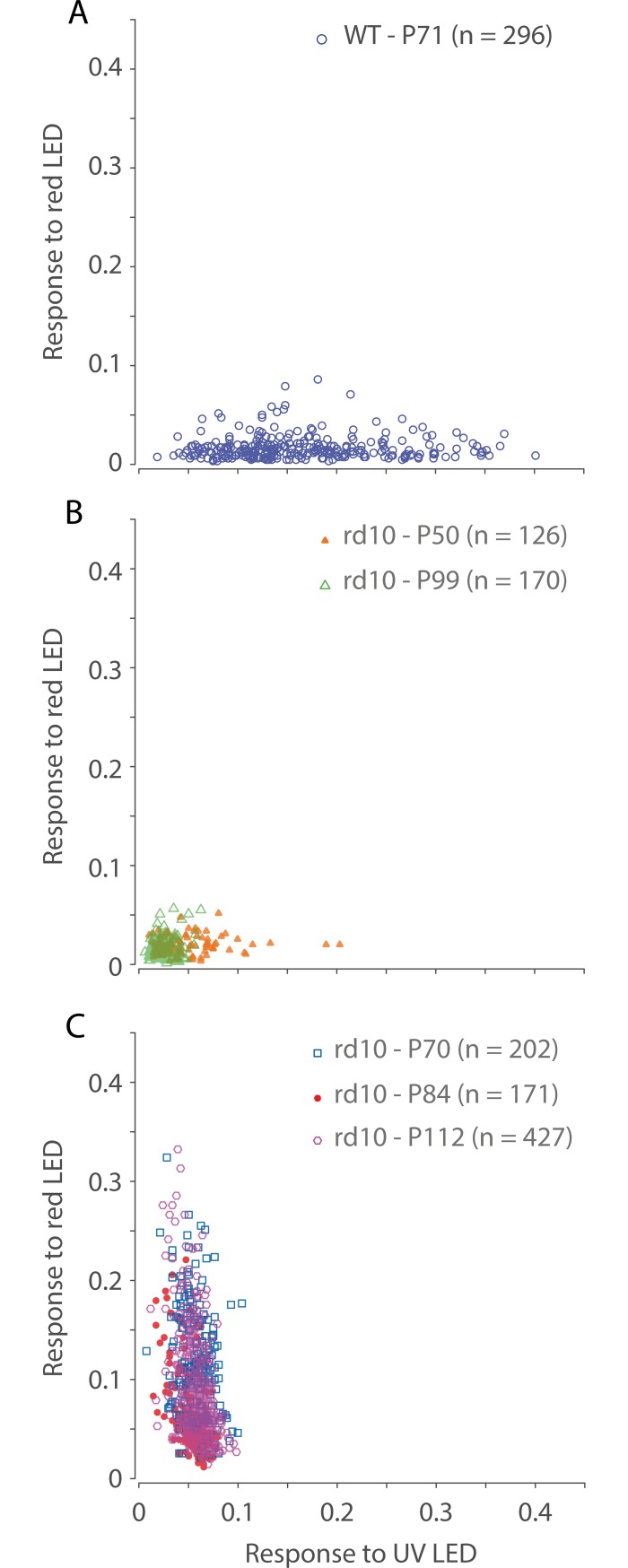
Single cell responses to visual stimulation. Scatter plot of single cell responses to UV (365 nm, 33.6 mW.cm^-2^ at the retina) and red (620 nm, 168.1 mW.cm^-2^ at the retina) flashing LED stimuli. Each data point represents one cell. (A) Recordings from WT mice injected with only GCaMP6s at age P71, 27 days after injection. (B) Recordings from two separate groups of *rd10* mice injected with only GCaMP6s. The first group was imaged at age P50, 26 days after injection, and the second group was imaged at age P99, 70 days after injection. (C) Recordings from one group of *rd10* mice injected with both GCaMP6s and ChrimsonR at three time points: P70, P84, and P112, which are 26, 40, and 68 days post injection respectively.

Fig 3 shows the process for extracting response time courses for single cells. A 365 nm LED was used to stimulate a WT mouse retina with a 0.2 Hz temporally modulated square-wave, 8° circular, uniform field. Three criteria were used to manually segment individual cells, a fluorescent intensity or “SUM” image (Fig 3A) computed by integrating all frames in the video, and functional maps (Fig 3B and 3C), constructed by applying a Fourier transform to the video data, pixel-by-pixel, to extract the power and phase of the GCaMP6s signal at the temporal frequency of the stimulus (F1). Fig 3B shows a response amplitude map, and Fig 3C shows a map of response phase. Cells with high fluorescent signal show up well in the SUM image. Cells that respond strongly to the visual stimulus, but do not necessarily have a high overall fluorescent signal, show up well in the response amplitude map. The map of response phase was used to further refine cell boundaries. Fig 3D shows the resulting cell segmentation mask. The segmentation mask was applied to the original video sequence and response time courses were computed for each segmented cell by averaging across all pixels in the segment. Response time courses of a neighboring ON cell and OFF cell are shown in Fig 3E and 3F, respectively. Three consecutive trials are shown for each cell. The growth in response for the trial shown in Fig 3F top row was consistently observed during the first trial as the retina adapted to light onset. S1 Movie shows an annotated version of the video data used to generate Fig 3. For presentation, frames from the original video file have been binned five frames per bin and playback is four times real speed. The stimulus waveform (bottom) and flashing square (top right) indicate when the LED was turned on. The response time course for each cell was subsequently analyzed in the Fourier domain to quantify responses to light stimulation (Fig 4). The F1 amplitude was divided by the mean signal (F0) to compute a normalized response.

### Wild-type mice exhibit robust responses to UV stimulation

Consistent with the spectral sensitivity of mouse photoreceptors, UV (365 nm LED) light stimulation drives vigorous responses in WT mice whereas red (620 nm LED) light stimulation does not. Light responses in WT mice are summarized in Fig 4A. The overall response to UV light was 0.16 ± 0.08 (mean ± SD) and to red light was 0.02 ± 0.01 (n = 296). Recording noise was analyzed by computing the mean and SD amplitude at frequencies from 10–12 Hz. We considered cells as significantly responding if the F1 amplitude was greater than the mean plus three SD of the noise. To UV light stimulation, of 306 cells analyzed, 305 cells had significant responses. To red light stimulation, of 351 cells analyzed, 170 cells had significant responses. A Rayleigh’s test of uniformity of response phase for all cells with significant responses to the red LED resulted in rejection of the null hypothesis (P < 0.01), that is, response phase to red light stimulation was not uniformly distributed. This indicates that in WT mice, there is a response to red light, albeit a small one. Data was pooled from 13 retinal locations across 4 eyes from 4 animals. Not all cells had responses recorded to both UV and red light stimulation, that is, data in Fig 4 shows the subset of data where cell responses to both UV and red light stimulation were recorded. Analyses of significant responses to either UV or red light, therefore have greater sample sizes.

### Substantial reduction of response in *rd10* mice by postnatal day 99

To show age dependent loss of visual responses in *rd10* mice, Fig 4B summarizes recordings in two groups of *rd10* mice that did not receive ChrimsonR aged postnatal day P50 and P99. Strong visual responses to UV light was observed in few cells at age P50, but responses declined by age P99. No robust responses to red light stimulation were observed at either age. The overall response for the P50 group to UV light was 0.04 ± 0.03 and red light 0.02 ± 0.01 (n = 126). The overall response for the P99 group to UV light was 0.03 ± 0.01 and red light 0.02 ± 0.01 (n = 170). At P50 and to UV light stimulation, of 130 cells analyzed, 129 showed significant responses. Analysis of response phase shows clustering of cells with ON and OFF responses (S2A Fig). At P50 and to red light stimulation, of 135 cells analyzed, 118 cells showed significant responses, and of these cells, response phase was significantly non-uniformly distributed (Rayleigh’s test p < 0.01, S2C Fig). At P99 and to UV light stimulation, of 174 cells analyzed, 128 showed significant responses. Analysis of response phase shows clustering of cells with only ON responses (S2B Fig). At P99 and to red light stimulation, of 199 cells analyzed, 87 cells showed significant responses, and of these cells, response phase was significantly non-uniformly distributed (Rayleigh’s test p < 0.01, S2D Fig). OFF responses must be mediated by photoreceptors. ON responses may be mediated by photoreceptors or by the intrinsic light sensitivity of melanopsin-containing ganglion cells. We conclude that *rd10* mice retain significant, although small, light responses at ages undetectable by electroretinogram [11, 12], but consistent with the findings of Stasheff et al. [38]. P50 data was pooled from 9 retinal locations across 2 eyes from 2 animals. P99 data was pooled from 11 retinal locations across 4 eyes from 3 animals.

### ChrimsonR restores lasting visual responses in *rd10* mice

Responses to UV and red light stimulation in *rd10* mice that received ChrimsonR are summarized in Fig 4C. Recordings were made in one group of mice at three different ages: P70, P84 and P112 (26, 40, and 68 days after injection of ChrimsonR respectively). Strong responses to red light stimulation were observed at all time points measured. The overall response to red light stimulation at P70 was 0.12 ± 0.06 (n = 202), at P84 0.07 ± 0.04 (n = 171), and at P112 0.09 ± 0.05 (n = 427). The overall response to UV light stimulation at P70 was 0.06 ± 0.02 (n = 202), at P84 0.05 ± 0.01 (n = 171), and at P112 0.06 ± 0.01 (n = 427). To red light stimulation, at P70, of 250 cells analyzed, 250 showed significant responses; at P84, of 172 cells analyzed, 170 showed significant responses; and at P112 of 487 cells analyzed, 487 showed significant responses. To UV light stimulation, at P70, of 216 cells analyzed, 215 showed significant responses; at P84, of 190 cells analyzed, 188 showed significant responses; and at P112 of 455 cells analyzed, 453 showed significant responses. The high proportion of cells responding significantly to UV light stimulation, with response amplitudes greater than those observed in *rd10* mice without ChrimsonR, may be due to the small, but not-insignificant, overlap of ChrimsonR action spectrum with the UV light spectrum. P70 data was pooled from 9 retinal locations across 4 eyes from 3 animals. P84 data was pooled from 7 retinal locations across 2 eyes from 2 animals; poor optical quality of the eye for one subject prevented imaging at this time point. P112 data was pooled from 10 retinal locations from 3 eyes across 3 animals.

**Fig 5:**
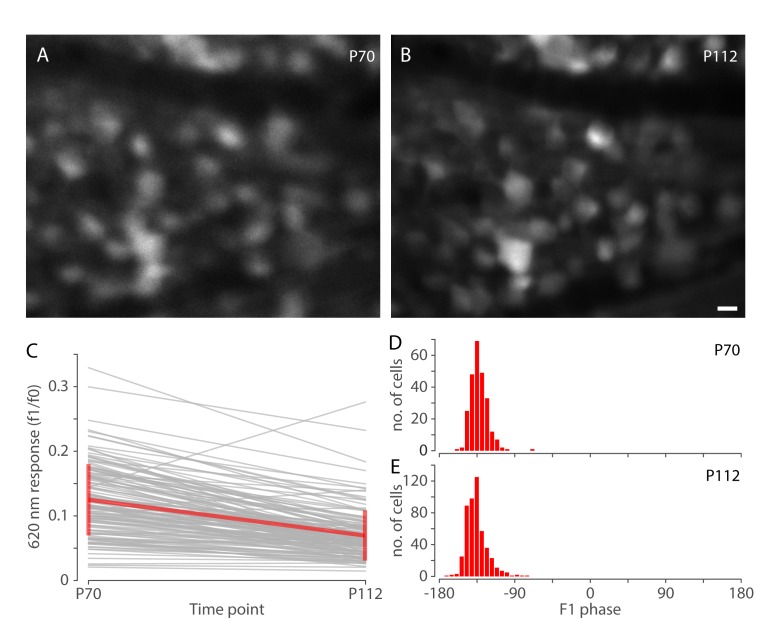
Tracking responses of individual cells over time in *rd10* mice with ChrimsonR. (A & B) Images of GCaMP6s fluorescent neurons in an *rd10* mouse eye treated with ChrimsonR imaged at two different time points, P70 (A) and P112 (B), at the same location. Scale bar in B indicates 10 μm. (C) Responses of individual cells (n = 137) to red (620 nm) light stimulation that were measured at both ages P70 and P112. Gray thin lines represent single cells. The thick red line shows the mean and SD. Data were pooled from seven retinal locations, from three animals. (D & E) Histograms of response (F1) phase for cells recorded in the same group of *rd10* ChrimsonR treated mice, at ages P70 (D) and P112 (E). The number of cells analyzed at P70 was 250, and at P112 was 487.

Because the FACILE method enables non-invasive neuronal recording in the living eye, the same cells from the same retinal locations in the same animal can be studied over time. Fig 5 shows analyses of responses measured in the same *rd10* mice with ChrimsonR at age P70 and P112. Fig 5A and 5B show images of GCaMP6s fluorescent neurons captured *in vivo* at age P70 and P112, respectively, for one retinal location. Fig 5C shows the responses for a subset of neurons (thin grey lines), from 7 locations across 3 animals, that were tracked across ages P70 and P112. The thick red line shows the mean, and error bars indicate SD. Despite the downward trend of response amplitudes to red light over time, analysis of response phase from all cells recorded at each time point (including cells that had responses recorded only at either P70 or P112, but not both) shows strong phase locking at both ages (Fig 5D) indicating significant, long-lasting responses to red light.

### ChrimsonR and GCaMP6s expression persists over many months

The optical quality of the mouse eye declines with age due to the development of cataracts and corneal opacities, which tend to form along the optical axis, to the point where adaptive optics imaging can no longer resolve individual cells. However, by imaging the retina with a fundus camera at an oblique angle, the opacities can be avoided allowing assessment of the long-term expression of GCaMP6s and ChrimsonR-tdTomato (S3 Fig). Expression of ChrimsonR-tdTomato and GCaMP6s in *rd10* mice remained highly visible out to P232, 188 days after injection. Expression remained widespread without overt changes in the pattern of fluorescence. Lack of drop out of fluorescent cells suggests that cell loss due to excessive expression of GCaMP6s or ChrimsonR-tdTomato, or phototoxicity from *in vivo* imaging (FACILE) was not an issue. The resting intensity of GCaMP6s intensity did, however, diminish slightly (S3A, S3C, S3E and S3F Fig).

## Discussion

Here we present an imaging method that stimulates and records the responses of many individual neurons in the intact eye. It combines high-resolution adaptive optics ophthalmoscopy of a genetically encoded calcium sensor (FACILE) with optogenetic activation of retinal neurons. The novel advantages of FACILE stem from its ability to non-invasively image neuronal function *in vivo*, and to do so repeatedly in the same animals over long periods of time. We show that recordings using FACILE can be used to track the loss of function in an animal model of retinal disease and evaluate the success of an optogenetic therapy for restoring vision. This new approach to all-optical stimulation and recording of individual neurons in the living eye is ideal for tracking long-term retinal function, as well as dissecting neuronal circuitry of the central nervous system.

### Tracking loss of function in retinal disease

We employed the *rd10* mouse model of retinal degeneration because it closely mimics the cause and pathogenesis of autosomal recessive photoreceptor degenerative disease in humans [11, 24]. Visual function in *rd10* mice measured with electroretinogram, peaks at 3 weeks of age before becoming non-detectable by 5 weeks to 2 months [11–13]. We first imaged *rd10* mice at age P99 expecting to find complete loss of visual responses by this late age. Response amplitudes to UV light stimulation were small (Fig 4B), however most cells analyzed showed significant responses. Analysis of response phase shows phase locking to the stimulus (S2B Fig). Nearly all responses were of ON type suggesting it may be mediated by melanopsin, as there is a small degree of overlap between the UV stimulus and melanopsin action spectrum (Fig 2). However, the density of M1 and M2 melanopsin containing ganglion cells in mouse is approximately 60 cells.mm^-2^ [39]. For our imaging field of view of 5 x 6.7° or 160 x 215 μm, the expected number of melanopsin positive cells is approximately two per imaging location, which is far fewer than the number of responding cells encountered. A third population of melanopsin containing ganglion cells exist, M3 type, but they are even more sparse, estimated to be ~10% of the total intrinsically photosensitive retinal ganglion cell population in rodents [40]. An alternative explanation may be, that despite the sparsity of melanopsin cells, their processes, which contain GCaMP6s, may be the source of the GCaMP6s signal. One method to test for this would be to perform the same experiment in *rd10* melanopsin knockout mice.

We subsequently measured the visual responses in P50 mice, an age where photoreceptor mediated light responses are expected to be absent [11–13], however see [38]. By recording from individual neurons, we found that nearly all cells examined with UV light stimulation showed significant responses. Responses were phased locked and clustered in two groups, ON and OFF cells (S2A Fig). OFF responses must be mediated by photoreceptor input. Taken together, the high spatial resolution of FACILE and sensitivity of the experimental design and analysis indicate that *rd10* mice retain photoreceptor mediated light responses for much longer than expected.

Photoreceptor degeneration in the *rd10* mouse begins several weeks after birth but progresses rapidly. The rhodopsin knockout (Rho^-/-^) mouse displays a much slower rate of photoreceptor degeneration, over months [41], and may be an alternative for tracking the loss of function with greater temporal detail.

FACILE can be used to study longitudinal retinal function at unprecedented resolution in a vast variety of animal models of retinal disease (for review see [42]). Glaucoma is a disease characterized by the degeneration of the RGCs and optic nerve and is a leading cause of irreversible vision loss worldwide [43–45]. Common pathologies have been found between Alzheimer’s disease and Glaucoma [46–48]. Inducible models of glaucoma such as elevated intraocular pressure or optic nerve crush are established [49]. Thus, understanding the functional changes in RGCs following axon injury using FACILE has the potential to improve the general understanding and treatment of many neurodegenerative diseases.

### Light levels to drive ChrimsonR

A novel aspect of this study of vision restoration is the use of ChrimsonR, which has an activation spectrum red-shifted by 45 nm relative to previous channelrhodopsins [18]. This is advantageous because light at longer wavelengths is safer than short wavelength light [19]. The maximum permissible exposure (MPE) as defined in the ANSI standard (2007, Z136, section 8) for human exposure to 620 nm up to 8 hours is 40 μW. The light level we employed to stimulate ChrimsonR in the mouse eye was 100 μW. To achieve an equivalent level of irradiance in the human eye, a scaling factor can be computed as the square of the ratio of the numerical apertures of mouse (0.49) and human (0.24) eyes, which yields 4.168 x 100 μW or 416.8 μW. Despite this high level of 620 nm light, we did not observe overt changes in retinal structure of drop out of fluorescent cells that might indicate phototoxicity. The aim of this study was to demonstrate the feasibility to track ChrimsonR mediated responses using *in vivo* imaging, therefore we used a high light level to drive a robust ChrimsonR response. Lack of drop out of fluorescent cells suggests that cell loss due to phototoxicity from *in vivo* imaging and ChrimsonR stimulation was not an issue (S3 Fig). Further studies are needed to determine the minimum light levels needed to stimulate ChrimsonR, in order to drive neuronal activity as well as visually guided behavior.

### Tracking the long-term efficacy of ChrimsonR vision restoration

This study demonstrates that light responses in retinal neurons of mice with photoreceptor degeneration can be with restored with ChrimsonR, which remains functional over a period of at least 6 weeks. The advantage of tracking responses of individual retinal neurons directly is to be able to measure any changes occurring at the single cell level. Using FACILE, despite ChrimsonR showing long-term function, we found there was an overall decrease in response amplitude from age P70 to P112. We do not know whether this decline is due to altered efficacy of GCaMP6 or responsivity of ChrimsonR. It has been previously reported that neurons with nuclear expression of GCaMP have attenuated fluorescent responses, perhaps due to impaired calcium homeostasis and GCaMP function [50]. However, both *in vivo* imaging and histology indicate that expression of GCaMP6s in neurons in this study was cytosolic.

Inserting ChrimsonR into inner retinal neurons such as ganglion cells restores visual responses that show limited functional diversity, that is, all responses are ON responses (Fig 5D and 5E). ChrimsonR, like other channelrhodopsins, is a light-gated, non-specific cation channel; cells expressing ChrimsonR depolarize in the presence of light and therefore are only able to produce “ON” responses. Inserting channelrhodopsin or halorhodopsin into bipolar cells [15, 51] or residual photoreceptor cell bodies [14] restores a greater diversity of light responses, for example ON and OFF responses, that are generated by intrinsic retinal circuitry. In future studies, we may target ChrimsonR to specific cell types to examine the diversity of visual responses that can be restored.

We propose that FACILE could help to accelerate the development of a wide variety of therapies for treating vision loss by allowing direct and repeatable visualization of cellular function over time throughout the therapeutic process. Such therapies include gene replacement, optogenetics, stem cell transplants and optoelectronic implants that do not preclude imaging of the retina.

### A new tool to accelerate retinal research and study neuronal circuitry

It is possible to record calcium responses of individual cells *in vivo* in the retina without AO [52, 53]. AO does allow substantial increases in resolution in all three spatial dimensions *in vivo* [26, 54], which will decrease the optical crosstalk from nearby cells. This in principle allows a cleaner signal from individual cells and probably increases the total number of cells from which recordings can be made. However, a quantitative assessment of the benefit of AO would require experiments we have not performed. A possible future application of FACILE is to image the calcium dynamics in subcellular compartments such as dendrites [55], or record from nearly every cell within a field of view with clarity [56].

A challenge with using intensity changes in a single-wavelength fluorescent sensor with FACILE is the difficulty in interpreting responses across individual cells, animals, and different time points. Expression level of the sensor is variable from cell to cell, even in local regions of the retina, and may change over time. In addition, variations in optical quality of the eye across imaging time points and finding the same plane of focus in repeated imaging sessions can result in variations in excitation intensity and fluorescent signal. The solution for these challenges of quantification lie in the use of fluorescence resonance energy transfer (FRET) based sensors [57] because they provide a ratiometric readout. FRET sensors can easily be combined with FACILE.

The FACILE method can be extended to study a multitude of biochemical and physiological processes within individual neurons using a wide array of fluorescent sensors, including molecules sensitive to voltage, glucose and glutamate, among others. The “*in vivo*” aspect makes FACILE well suited for studies in large animals such as dogs and non-human primates because it reduces the number of animals needed when assessing the time course of vision loss and therapy.

The read-write capability we demonstrate here in the mouse retina is a first step toward the development of a host of new tools for dissecting the neuronal circuits. Selective targeting of ganglion cell subtypes with specific promoters is already possible. For example, a comprehensive characterization of some ganglion cell subtypes such as alpha-like, On-Off and On-type direction-selective ganglion cells has been facilitated by transgenic mouse lines which express fluorescent proteins under the control of specific promoters [58–63]. Combined with the vast range of reporter molecules already available, it looks increasingly likely that the most successful method to interface the nervous system with computers will be an optical one.

## Supporting information

S1 Fig Excitation and emission spectra of GCaMP6s and tdTomato. Action spectra plot showing GCaMP6s excitation and emission, measured spectrum of 488 nm laser source for exiting GCaMP6s, band-pass filter imaging GCaMP6s fluorescence, and tdTomato excitation and emission.
(EPS)

S2 Fig Response phase to UV and red light stimulation in *rd10* mice. Histograms of response phase to UV (365 nm) and red (620 nm) light stimulation for cells with significant responses recorded in two different groups of rd10 mice, at age P50 (A & C) and P99 (B & D). Both groups were injected only with GCaMP6s. Number of cells analyzed in the P50 group were 129 for UV light stimulation and 118 for red light stimulation, and at P99 were 128 for UV light stimulation and 87 for red light stimulation.
(EPS)

S3 Fig Long-term expression of ChrimsonR and GCaMP6s in *rd10* retinae. Fundus images from two rd10 mouse eyes (A-D and E-F) imaged at ages P93 (49 days after injection; A, B, E, F) and P232 (188 days after injection; C, D, G, H) showing persistent fluorescence from GCaMP6s (A, C, E, G) and Chrimson-tdTomato (B, D, F, G). Panels E and F are the same as in Fig 1C and 1D. Scale bar in h indicates 500 μm and applies to all panels.
(EPS)

S1 Movie Adaptive optics calcium imaging of RGC activity in the living mouse eye. Responses of RGCs in the living WT mouse eye to a flashing uniform field 365 nm LED stimulus visualized using adaptive optics imaging of GCaMP6s fluorescence. For presentation, frames are binned 5:1 and playback speed is increased fourfold. The stimulus waveform (bottom) and flashing square (top right) indicate when the LED was on.
(MP4)
